# Preliminary analysis of AI-based thyroid nodule evaluation in a non-subspecialist endocrinology setting

**DOI:** 10.1007/s12020-025-04287-8

**Published:** 2025-06-05

**Authors:** Pablo Fernández Velasco, Lucia Estévez Asensio, Beatriz Torres, Ana Ortolá, Emilia Gómez Hoyos, Esther Delgado, Daniel de Luís, Gonzalo Díaz Soto

**Affiliations:** 1 Department of Endocrinology and Nutrition, Hospital Clínico Universitario Valladolid, Valladolid, Spain; 2 Centro de Investigación de Endocrinología y Nutrición Clínica (CIENC), Facultad de Medicina, Universidad de Valladolid, Valladolid, Spain

**Keywords:** Thyroid nodule, Artificial intelligence, AI-DSS, Ultrasound, ACR TI-RADS, ATA guidelines, Risk stratification, General endocrinology

## Abstract

**Purpose:**

Thyroid nodules are commonly evaluated using ultrasound-based risk stratification systems, which rely on subjective descriptors. Artificial intelligence (AI) may improve assessment, but its effectiveness in non-subspecialist settings is unclear. This study evaluated the impact of an AI-based decision support system (AI-DSS) on thyroid nodule ultrasound assessments by general endocrinologists (GE) without subspecialty thyroid imaging training.

**Methods:**

A prospective cohort study was conducted on 80 patients undergoing thyroid ultrasound in GE outpatient clinics. Thyroid ultrasound was performed based on clinical judgment as part of routine care by GE. Images were retrospectively analyzed using an AI-DSS (Koios DS), independently of clinician assessments. AI-DSS results were compared with initial GE evaluations and, when referred, with expert evaluations at a subspecialized thyroid nodule clinic (TNC). Agreement in ultrasound features, risk classification by the American College of Radiology Thyroid Imaging Reporting and Data System (ACR TI-RADS) and American Thyroid Association guidelines, and referral recommendations was assessed.

**Results:**

AI-DSS differed notably from GE, particularly assessing nodule composition (solid: 80%vs.36%,p < 0.01), echogenicity (hypoechoic:52%vs.16%,p < 0.01), and echogenic foci (microcalcifications:10.7%vs.1.3%,p < 0.05). AI-DSS classification led to a higher referral rate compared to GE (37.3%vs.30.7%, not statistically significant). Agreement between AI-DSS and GE in ACR TI-RADS scoring was moderate (r = 0.337;p < 0.001), but improved when comparing GE to AI-DSS and TNC subspecialist (r = 0.465;p < 0.05 and r = 0.607;p < 0.05, respectively).

**Conclusion:**

In a non-subspecialist setting, non-adjunct AI-DSS use did not significantly improve risk stratification or reduce hypothetical referrals. The system tended to overestimate risk, potentially leading to unnecessary procedures. Further optimization is required for AI to function effectively in low-prevalence environment.

## Introduction

Thyroid nodule pathology is a highly prevalent condition in daily clinical practice, with an estimated incidence of up to 60% in ultrasound-based population studies (1,2). Thyroid ultrasound is the imaging modality of choice for evaluating thyroid nodules, allowing for morphological characterization and guiding diagnostic and therapeutic decisions [1, 2]. Although most thyroid nodules are benign, a small percentage are malignant [3], highlighting the need for accurate and standardized evaluation to avoid unnecessary procedures in clinically irrelevant lesions while preventing the underdiagnosis of malignant lesions that may impact patient morbidity and mortality.

Currently, the ultrasound-based approach to thyroid nodule pathology relies on risk stratification scales. These scales aim to standardize clinical management by minimizing the subjectivity inherent in ultrasound assessment, which remains one of its main limitations [4, 5]. However, evaluating the descriptors that constitute these scales, such as echogenicity, composition, margins, presence of echogenic foci, or the anteroposterior-to-transverse ratio of the nodule, still involves a significant degree of subjectivity. In fact, several studies report moderate or even low interobserver agreement in characterizing these descriptors [6, 7]. Thus, diagnostic and therapeutic decisions remain highly dependent on the experience and expertise of the ultrasound operator at a given time.

Recently, artificial intelligence (AI) has emerged as a promising tool for improving diagnostic accuracy in various fields of thyroidology [8]. Its application in thyroid ultrasound has gained increasing interest, with the development of deep learning algorithms capable of identifying suspicious nodules in controlled validation settings with accuracy comparable to that of expert endocrinologists or radiologists [9]. Moreover, AI-based decision support systems (AI-DSS) have demonstrated an overall improvement in the diagnostic capability of thyroid ultrasound, enhancing the diagnostic precision of evaluators and reducing interobserver variability in real-world clinical practice in high-expertise thyroid nodule clinics (TNC) with relatively low malignancy prevalence [10]. However, no studies to date have analyzed the utility of AI in settings with limited ultrasound expertise and very low malignancy prevalence. In this context, the primary objective of the ultrasound evaluation is to screen for thyroid nodule pathology that may require additional procedures in subspecialized units for further assessment. This initial evaluation is crucial, as up to one-third of patients referred to these highly subspecialized thyroid nodule pathology units do not require additional studies or invasive procedures [11].

The present study aimed to assess the impact of using an AI-DSS on the ultrasound interpretation of thyroid nodules and its influence on risk stratification according to the American College of Radiology Thyroid Imaging Reporting and Data System (ACR TI-RADS) and American Thyroid Association guidelines (ATA) categorization in a real-world cohort of patients evaluated in routine clinical practice by general endocrinologists (GE) without subspecialty thyroid imaging training. Additionally, the study analyzed the agreement between the ultrasound characteristics defined by observers and those generated by the AI-DSS, as well as the potential impact of the system on modifying referrals to subspecialized TNC if the system had been used.

## Material and Methods

A prospective cohort study was conducted on the first 80 consecutive patients aged over 18 who were referred to general endocrinology outpatient consultations and required clinical ultrasound screening based on medical criteria (Point-of-Care Ultrasound, POCUS) between March and December 2023.

Demographic, clinical, and biochemical data were prospectively collected, including age, sex, personal and family history of thyroid pathology, age at diagnosis, diagnostic method, overall thyroid and nodular image characteristics, and serum TSH and free thyroxine (T4) levels. Ultrasound images were obtained in DICOM format, including transverse and longitudinal views. Patients who did not provide consent or whose ultrasound images were of insufficient quality due to poor resolution, artifacts, or non-standardized acquisition were excluded.

Ultrasound examinations were performed by five GE without subspecialized training in thyroid imaging, as part of their routine clinical practice in the general endocrinology outpatient clinic, within the context of screening prior to referral to TNC. In cases where POCUS identified a previously unevaluated thyroid nodule, its ultrasound characteristics were systematically recorded by the clinicians using a standardized data sheet, following the classification criteria of the ACR TI-RADS and the American Thyroid Association Guidelines 2015 [1, 4]. Based on their clinical judgment, cases requiring additional procedures, such as follow-up or fine-needle aspiration biopsy (FNA), were referred to the TNC for subspecialized evaluation. Nodules ultimately referred to the TNC were reassessed prospectively by one of two endocrinologists, each with more than 15 years of expertise in thyroid ultrasound and minimally invasive techniques, who systematically collected the same information for definitive analysis, exclusively following the ACR TI-RADS evaluation for clinical decision-making.

**Fig. 1:**
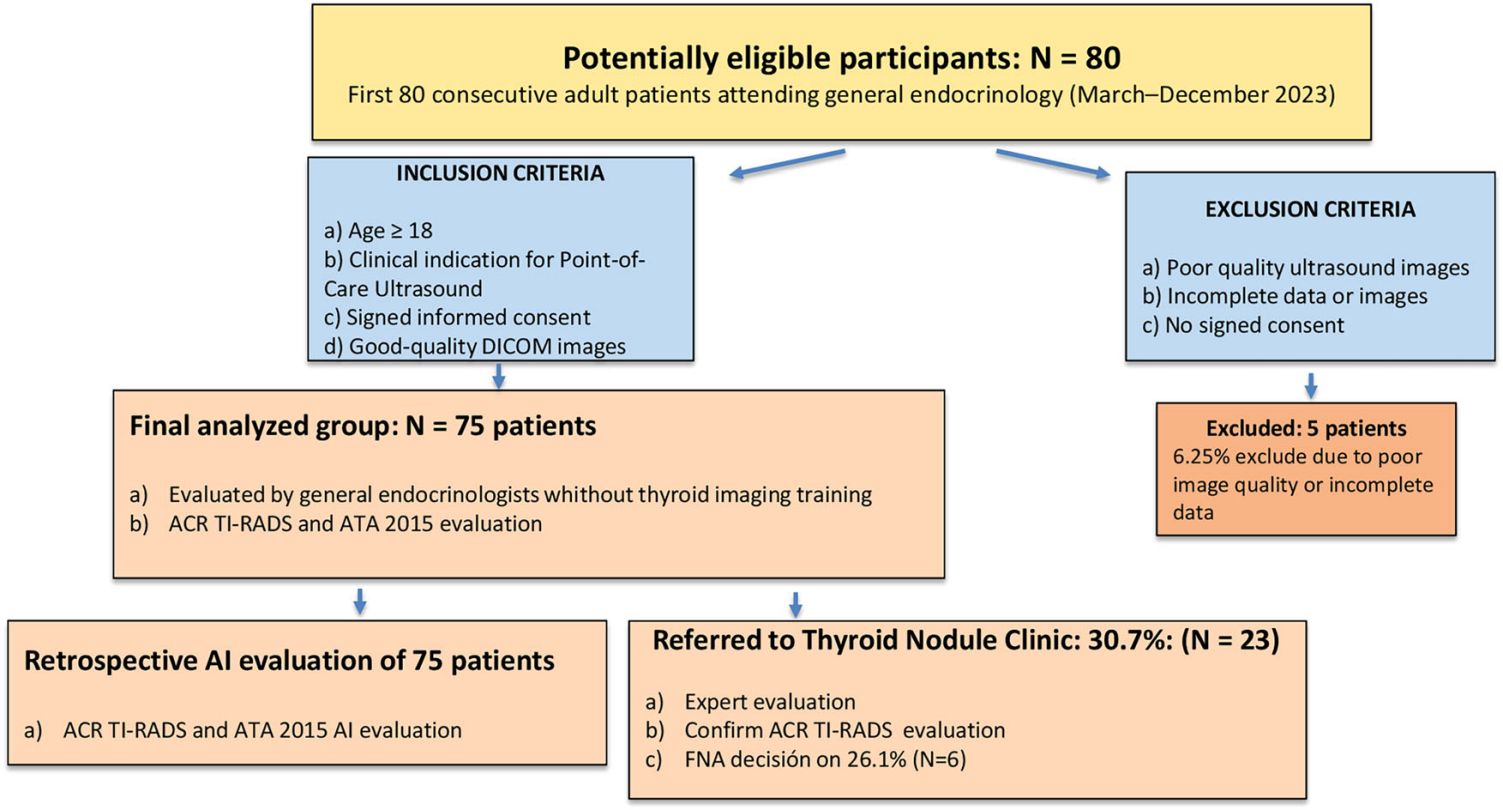
Flowchart showing inclusion/exclusion criteria and evaluations by general endocrinologists, thyroid nodule clinic subspecialists, and the AI-based decision support system

The thyroid nodules previously evaluated in the GE consultation were retrospectively analyzed using an AI-DSS Koios DS, NY, USA. The ultrasound characteristics described by the endocrinologists with and without subspecialized imaging training were compared with those automatically generated by the AI-DSS, assessing the level of agreement between both evaluations. Additionally, the AI-DSS ultrasound categorization of each thyroid nodule, risk assessment, and its recommendation regarding referral to the TNC—based on AI-suggested follow-up or FNA—were evaluated and compared to the recommendations made by GE (Fig. 1).

Ultrasound images were acquired in clinical practice using a Toshiba Xario 100 ultrasound machine with a multifrequency linear probe. A standardized protocol was established, including the capture of at least two orthogonal images per nodule to ensure optimal representation of its morphological characteristics. Image selection was performed by an endocrinologist with subspecialized ultrasound imaging training, excluding those with poor diagnostic quality.

### AI-based DSS

The AI-DSS employed in this study is an FDA-approved AI-based decision support system designed to assist in the evaluation of thyroid nodules through ultrasound imaging based on computer vision and machine learning techniques for the automated evaluation of ultrasound images. Thyroid nodule categorization was performed according to the descriptors outlined in both ACR TI-RADS and ATA guidelines, with the exception of extrathyroidal extension (4). Additionally, the system incorporated an AI Adapter module, which allows for risk stratification adjustments within a −2 to +2 point range, with the objective of optimizing ACR TI-RADS and ATA classification. Based on the final adjusted score, the system generated clinical recommendations, including the indication for FNA or follow-up, in accordance with size criteria and risk scoring thresholds established in the ACR TI-RADS and ATA 2015 guidelines [1, 4].

### Statistical analysis

The required sample size was calculated for a comparison of proportions based on the expected classification rate of ACR TI-RADS < 3 by the GE and by the AI-DSS. We assumed a rate of 45% for the GE assessment and 75% for the AI-DSS, with a statistical power of 90% and a 95% confidence level (α = 0.05), resulting in a required sample size of 58 nodules.

Quantitative variables were described as mean ± standard deviation (SD) for normally distributed data, while non-parametric variables were expressed as median and interquartile range (IQR). Normality was assessed using the Kolmogorov-Smirnov test. Differences between groups were analyzed using Student’s t-test for continuous variables with normal distribution and Friedman and Wilcoxon tests for non-parametric data. Categorical variables were expressed as percentages (%) and compared using the chi-square test, applying Fisher’s exact test when necessary.

Interobserver agreement was assessed using Cohen’s kappa coefficient, analyzing reproducibility in the classification of thyroid nodule ultrasound characteristics and their categorization according to the ACR TI-RADS and ATA systems.

The correlation between quantitative variables was evaluated using scatter plots and Pearson’s correlation coefficient. Statistical significance was set at p < 0.05. All statistical analyses were performed using IBM SPSS Statistics® version 29.0.2.0 (SPSS Inc., Chicago, IL, USA).

All patients provided written informed consent prior to their inclusion in the study. The protocol was approved by the Clinical Research Ethics Committee of the Hospital Center (PI 23-3198), and the study was conducted in accordance with the principles of the Declaration of Helsinki

## Results

A total of 80 patients were initially enrolled, of whom 75 were included in the final analysis following the exclusion of 6.25% of cases due to poor image quality or incomplete imaging data (Fig. 1). The mean age at diagnosis was 59.7 ± 14.1 years, with a predominance of female patients (94.7%). A total of 22.7% of patients had a family history of goiter, while 18.7% had a history of functional thyroid disorder. The most frequently employed diagnostic method was thyroid ultrasound (49.3%), followed by the detection of a palpable nodule (22.7%). The right thyroid lobe was the most commonly affected (57.3%). Among the patients assessed via POCUS, 30.7% were referred to the TNC for subspecialized evaluation at the discretion of the GE (Table 1).

**Table 1:** Clinical and Biochemical Characteristics of the Analyzed Population

BASIC CHARACTERISTICS	MEAN ± SD / PERCENTAGE
Number of patients	75
Percentage of women	94.7%
Age (years)	59.7 ± 14.1
**Personal History (%)**	
• Head and neck radiotherapy	2.7%
• Oncologic disease	1.3%
• Iodized salt intake	20%
**Family History (%)**	
• Thyroid cancer	1.3%
• Goiter	22.7%
• Hyper/Hypothyroidism	18.7%
**TSH (µUI/mL)**	2.2 ± 2.1
**Free T4 (ng/dL)**	1.3 ± 0.4
**Free T3 (pg/mL)**	4.4 ± 2.5
**Thyroid peroxidase antibody (UI/mL)**	99.7 ± 138.4
**Levothyroxine dose (µg/day)**	75 ± 34
**Diagnosis method (%)**	
• Palpable nodule	22.7%
• Incidental finding (Computed Tomography Scan)	5.3%
• Previous cervical ultrasound	49.3%
• Compressive symptoms	1.3%
• Gland size increase	2.7%
• Functional alteration	17.3%
• Positron Emission Tomography scan	1.3%
**Right laterality (%)**	57.3%
**Global thyroid echogenicity (%)**	
• Homogeneous	72%
• Heterogeneous	16%
• Thyroiditis	12%
**Nodule dimensions (mm)**	
• Anteroposterior diameter	15.2 ± 9
• Transverse diameter	11.3 ± 6.1
• Longitudinal diameter	18.5 ± 11.4
**Referred to Thyroid Nodule Clinic (%)**	30.7%

Table 2 presents the ultrasound characteristics of thyroid nodules evaluated in GE practice, as assessed by the AI-DSS and those ultimately referred to the TNC. When comparing AI-DSS versus GE assessment, significant differences were observed in nodule composition, echogenicity, presence of echogenic foci, ACR TI-RADS classification, and ATA classification.

**Table 2:** Descriptive characteristics of thyroid nodules evaluated by GE, AI-DSS, and TNC subspecialist

Characteristics (%)	General Endocrinologist (n = 75)	AI-DSS (n = 75)	TNC (30,7% cases) (n = 23)
Wider than tall	93,3 (n = 70)	77,3 (n = 58)	91,3 (n = 21)
Composition*			
Sólid	36 (n = 27)	80 (n = 60)	73,9 (n = 75)
Solid-cystic	21,3 (n = 16)	16 (n = 12)	8,7 (n = 2)
Spongiform	38,7 (n = 29)	1,3 (n = 1)	8,7 (n = 2)
Cystic	4 (n = 3)	2,7 (n = 2)	8,7 (n = 2)
Echogenicity*			
Very hypoechoic	1,3 (n = 1)	14,7 (n = 11)	
Hypoechoic	14,7 (n = 11)	37,3 (n = 28)	18,2 (n = 4)
Iso/Hyperechoic	57,3 (n = 43)	46,7 (n = 35)	77,3 (n = 17)
Anechoic	26,7 (n = 20)	1,3 (n = 1)	4,5 (n = 1)
Margins			
Well-defined	90,7 (n = 68)	94,7 (n = 71)	95,2 (n = 20)
Lobulated/Irregular	4 (n = 3)	5,3 (n = 4)	4,8 (n = 1)
Not visible	5,3 (n = 4)		
Echogenic foci*			
Comet-tail artifact	21,3 (n = 16)	1,3 (n = 1)	
Macrocalcifications	17,3 (n = 13)	4 (n = 3)	22,7 (n = 5)
Rim calcifications	2,7 (n = 2)	1,3 (n = 1)	
Microcalcifications	1,3 (n = 1)	10,7 (n = 8)	4,5 (n = 1)
None	57,3 (n = 43)	76 (n = 57)	72,7 (n = 16)
ACR TI-RADS ≤ 3*	77 (n = 58)	44 (n = 33)	65 (n = 15)
Mean TI-RADS points ± SD	2,4 ± 2.0	4,4 ± 4.6	3,2 ± 1.0
ATA*			
Benign	22,7 (n = 17)	1,6 (n = 1)	
Very low suspicion	32 (n = 24)	4,8 (n = 4)	
Low suspicion	29,3 (n = 2)	4,6 (n = 3)	
Intermediate suspicion	12 (n = 9)	7,9 (n = 6)	
High suspicion	4 (n = 3)	39,7 (n = 30)	
**FNA (%)**	**30,7 (n** = **23)**	**37,3 (n** = **28)**	**24,6 (n** = **6)**

*p < 0.05 for comparison between General Endocrinologist and AI-DSS

According to the GE, the most frequent nodule composition was spongiform, which was significantly more common than in the AI-DSS evaluation (38.7% vs. 1.3%, p < 0.05). Similarly, GE classified a significantly lower proportion of nodules as solid compared to AI-DSS (36.0% vs. 80.0%, p < 0.05). Regarding echogenicity, an iso/hyperechoic appearance was the most frequent feature in both GE and AI-DSS (57.3% vs. 46.7%, respectively). Conversely, GE identified significantly fewer hypoechoic nodules than AI-DSS (16.0% vs. 52.0%, p < 0.05). Well-defined margins were reported in over 90% of cases by both GE and AI-DSS (90.7% vs. 94.7%, not statistically significant (ns)). However, for echogenic foci, GE more frequently reported comet-tail artifacts than AI-DSS (21.3% vs. 1.3%, p < 0.05), and identified a lower proportion of microcalcifications (1.3% vs. 10.7%, p < 0.05).

Regarding ACR TI-RADS stratification, a significantly greater proportion of nodules were classified as ACR TI-RADS ≤ 3 by GE compared to AI-DSS (77.0% vs. 44.0%, p < 0.05), with a lower mean score assigned by GE (2.4 ± 2.0 vs. 4.4 ± 4.6, ns). In terms of patient referrals, 30.7% of cases were referred to the TNC by GE, whereas AI-DSS recommended additional procedures in 37.3% of cases (Table 2)

Within the subgroup of patients referred to the TNC (30.7% of the cohort), 73.9% of nodules were solid, and 77.3% displayed iso- or hyperechoic features. Well-defined margins were predominant (95.2%), whereas echogenic foci were minimally present. In terms of ACR TI-RADS stratification, 65% of nodules in this subgroup were classified as TI-RADS ≤ 3, with a mean score of 3.2 ± 1.0 points. Ultimately, FNA was performed in 24.6% of the nodules evaluated within the TNC, and in all cases, Bethesda II cytological findings confirmed benign pathology.

Furthermore, interobserver agreement in thyroid nodule characterization between the GE, the TNC subspecialist, and AI-DSS was evaluated (Table 3). A low level of agreement was observed between AI-DSS and the GE for most ultrasound characteristics, whereas agreement improved when comparing AI-DSS with the TNC, particularly in the evaluation of nodule composition (Kappa = 0.352, p < 0.05), echogenicity (Kappa = 0.511, p < 0.05), and presence of echogenic foci (Kappa = 0.405, p < 0.05). Notably, margin evaluation demonstrated perfect agreement between AI-DSS and the TNC subspecialist (Kappa = 1.000). Conversely, classification based on ACR TI-RADS demonstrated poor agreement with the GE (Kappa = 0.026, not significant), while agreement improved slightly with the TNC (Kappa = 0.123, p < 0.05). Similarly, ATA classification did not show significant concordance with the GE (Kappa = 0.012, not significant).

**Table 3:** Concordance (Cohen’s kappa coefficient) in thyroid nodule ultrasound evaluation between the GE, TNC subspecialist, and AI-DSS

Characteristics	General Endocrinologist vs AI	p-value	TNC vs AI	p-value	General Endocrinologist vs TNC	p-value
Wider than tall	0.189	<0.05	0.181	<0.05	0.187	<0.05
Composition	0.226	<0.05	0.352	<0.05	0.495	<0.05
Echogenicity	0.107	ns	0.511	<0.05	0.637	<0.05
Margins	0.051	ns	1.000	ns	0.294	<0.05
Echogenic foci	0.180	<0.05	0.405	<0.05	0.472	<0.05
ACR-TIRADS category	0.026	ns	0.123	<0.05	0.322	<0.05
ATA classification	0.012	ns	–	–	–	–

*ns* not statistically significant

Similarly, the agreement between the GE and the TNC was analyzed (Table 3). A moderate-to-high level of agreement was observed in the evaluation of echogenicity (Kappa = 0.637, p < 0.05), composition (Kappa = 0.495, p < 0.05), and echogenic foci (Kappa = 0.472, p < 0.05). The assessment of margins showed lower agreement (Kappa = 0.294, p < 0.05), whereas the evaluation of the taller-than-wide ratio demonstrated the lowest agreement (Kappa = 0.187, p < 0.05), though still statistically significant. Finally, regarding ACR TI-RADS classification, the observed agreement was moderate (Kappa = 0.322, p < 0.05).

The level of agreement in thyroid nodule classification was further assessed through a correlation analysis of the ACR TI-RADS scores assigned by the GE, the TNC, and the AI-DSS. A weak correlation was found between the GE and AI-DSS scores (r = 0.337; p < 0.001), while the correlation between the TNC subspecialist and AI-DSS was moderate (r = 0.465; p < 0.05). On the other hand, the highest level of agreement was observed between the GE and the TNC, demonstrating a strong correlation (r = 0.607; p < 0.05).

A comparison was made between the characteristics of the nodules analyzed by the GE that were or were not referred for evaluation by the TNC, identifying significant differences in multiple variables (Table 4). The nodules referred to the TNC were larger in all three analyzed dimensions: anteroposterior diameter (20 ± 11.2 mm vs. 13 ± 6.8 mm, p < 0.01), transverse diameter (14.3 ± 6.6 mm vs. 9.9 ± 5.4 mm, p < 0.01), and longitudinal diameter (25.6 ± 13.5 mm vs. 15.4 ± 8.8 mm, p < 0.01). Likewise, the mean score on the ACR TI-RADS scale was significantly higher in nodules referred to the TNC (3.8 ± 2.2 vs. 1.8 ± 1.6 points, p < 0.01). Regarding composition and echogenicity, referred nodules had a higher proportion of solid pattern (60.9% vs. 25%, p < 0.01) and a lower frequency of anechoic echogenicity (4.3% vs. 36.5%, p < 0.05). Additionally, the overall gland echogenicity showed a heterogeneous pattern in 47.8% of referred cases, compared to 19.2% in non-referred cases (p < 0.05).

**Table 4:** Comparison of characteristics between nodules referred or not referred to TNC

Characteristics	Non-TNC	TNC	p-value
**Anteroposterior diameter (mm)**	13 ± 6.8	20 ± 11.2	<0.01
**Transverse diameter (mm)**	9.9 ± 5.4	14.3 ± 6.6	<0.01
**Longitudinal diameter (mm)**	15.4 ± 8.8	25.6 ± 13.5	<0.01
**Mean ACR TI-RADS points**	1.8 ± 1.6	3.8 ± 2.2	<0.01
**Wider than tall (%)**	98.1%	82.6%	<0.05
**Global thyroid echogenicity (%)**			
Thyroiditis/Heterogeneous	19.2%	47.8%	<0.05
**Composition (%)**			
Solid	25%	60.9%	<0.01
**Echogenicity (%)**			
Anechoic	36.5%	4.3%	<0.05
**ATA classification (%)**			
Intermediate & high suspicion	5.7%	39.1%	<0.01
**ACR TI-RADS classification (%)**			
ACR TI-RADS 4-5	13.5%	43.5%	<0.01

Regarding risk stratification systems, 39.1% of referred nodules were classified as intermediate or high suspicion according to ATA (39.1% vs. 5.7%, p < 0.01), while 43.5% of the referred nodules were categorized as ACR TI-RADS 4 or 5 (43.5% vs. 13.5%, p < 0.01). Finally, the wider-than-tall shape was less frequent in referred nodules (82.6% vs. 98.1%, p < 0.05).

**Fig. 2:**
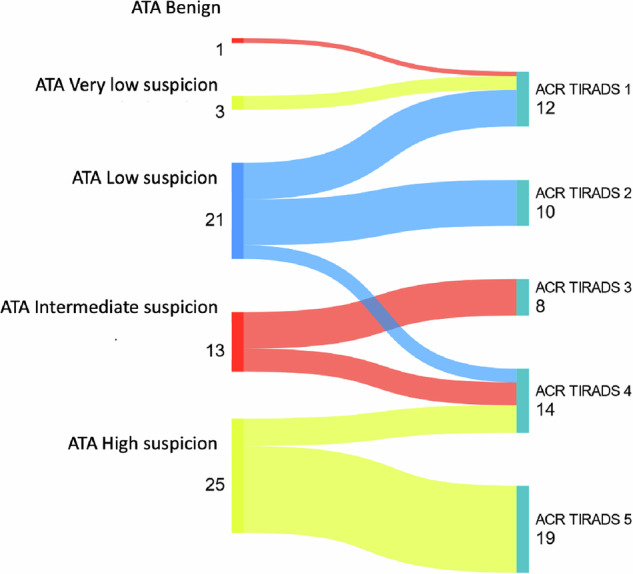
Classification of nodules as ATA and ACR TI-RADS using the AI-DSS

Finally, differences in the classification of thyroid nodules between the ATA 2015 and ACR TI-RADS systems were analyzed when performed by the AI-DSS. It was observed that nodules classified as low, intermediate, or high suspicion according to ATA 2015 were reclassified into a different risk category in ACR TI-RADS in 31.9% of cases (Fig. 2).

## Discussion

The present study evaluated the impact of an AI-based diagnostic support system on the ultrasound assessment of thyroid nodules in a screening setting conducted by specialists without specific training in thyroid imaging, within a very low malignancy prevalence context. To date, studies on these systems have focused on subspecialist environments or have been conducted by experts, either in training or with experience [10, 12]. However, their utility in low-complexity settings, where thyroid nodules are predominantly benign, has not been explored.

Our results indicate that using AI-DSS independently of clinician evaluation does not significantly improve diagnostic accuracy, optimize risk stratification, or reduce the number of referrals for additional studies in subspecialized units. In fact, this study observed low agreement between the evaluations performed by GE and the AI-DSS alone, particularly in key risk descriptors such as composition, echogenic foci, and echogenicity. Overall, the AI-DSS adopted a conservative approach when classifying nodules, leading to a higher number of follow-up or FNA recommendations compared to the clinical evaluation by the GE.

In recent years, various AI models have demonstrated diagnostic performance comparable to that of expert endocrinologists and radiologists in thyroid nodule ultrasound assessment, achieving similar sensitivity and specificity in risk classification according to scales such as ACR TI-RADS [13]. The AI algorithm analyzed in this study has been shown to reduce interobserver variability and optimize decision-making, decreasing unnecessary FNAs without compromising the detection of malignant nodules [10, 12, 14]. However, these studies were conducted in highly subspecialized centers, with the participation of clinicians with advanced training in thyroid imaging, where malignancy prevalence was relatively high [10, 12, 14]. This raises questions about the applicability of this AI-DSS in a setting with lower thyroid ultrasound expertise and low malignancy prevalence, where the main goal is screening and referral rather than immediate risk stratification.

In this scenario, AI could serve as a potential support tool to improve workflow and optimize referrals to subspecialized units. To our knowledge, this is the first study to evaluate the performance of an AI-DSS in a real-world low-complexity clinical environment, providing autonomous performance data outside of subspecialized reference centers or validation studies.

Significant differences were observed in thyroid nodule classification between GE, AI-DSS, and TNC evaluation, particularly in nodule composition, echogenicity, and echogenic foci. While general GE classified 38.7% of nodules as spongiform, AI-DSS assigned this category to only 1.3%, despite the fact that spongiform nodules are virtually always benign [1]. Furthermore, AI-DSS classified 52% of nodules as hypoechoic or very hypoechoic, compared to 16% reported by GE. Regarding echogenic foci, AI detected comet tail artifacts in only 1.3% of cases, whereas GE reported them in 21.3% of cases, which has a substantial impact on final risk categorization. Similarly, when classified using ATA guidelines, AI-DSS reduced the proportion of nodules categorized as benign (1.6% vs. 22.7% by GE) and increased high-suspicion classifications (39.7% vs. 4%). These results further confirm that AI-DSS follows a more conservative approach, tending toward risk overestimation.

Several plausible explanations exist for the observed discrepancies. These include technical characteristics of ultrasound imaging, the ultrasound equipment used, non-standardized image settings, the static nature of analyzed images, and the clinical environment for which AI-DSS was trained. Although Koios DS is designed to process DICOM-format images and is compatible with various ultrasound devices, its performance may be affected by image quality and settings [15]. In this study, images were obtained by endocrinologists without specific imaging expertise, though they routinely used ultrasound as a screening tool in daily practice. Additionally, pre-study training was conducted to ensure correct image acquisition. This scenario is comparable, if not superior, to what would be encountered in general medicine settings. Furthermore, the static nature of the images may have hindered echogenic foci assessment, particularly in differentiating microcalcifications from comet tail artifacts. This limitation is common to most commercially available AI systems, regardless of the environment in which they are tested. Despite previous studies demonstrating that AI-DSS performance is comparable to that of a subspecialist in thyroid nodule evaluation, guidelines recommend its use as an adjunctive tool. Our findings reinforce the importance of considering AI-DSS as a complementary tool within clinical evaluation, especially in low-complexity environments dominated by benign nodules, rather than as a replacement for physician assessment.

The Koios DS regulatory framework emphasizes that its use should serve as an additional support to clinical evaluation by trained endocrinologists, without replacing human diagnostic interpretation [10, 12]. However, the risk of fully delegating ultrasound assessment to AI systems remains a concern, particularly in settings with limited training in thyroid nodule pathology. Similarly, prioritization of nodules requiring further investigation in subspecialized units is a key factor that AI systems must address to ensure optimal performance.

Thyroid ultrasound is primarily a screening tool, designed to rule out malignancy and minimize the need for invasive procedures, ensuring a high negative predictive value. Indeed, new guidelines and classification systems aim to reinforce the reduction of procedures, minimizing overdiagnosis and overtreatment of differentiated thyroid carcinoma [1, 16]. Previous studies conducted in high-expertise settings with greater malignancy prevalence have demonstrated that AI-DSS can improve diagnostic accuracy and reduce interobserver variability [10, 12]. However, in our study, conducted in a real-world clinical environment with very low malignancy prevalence, AI-DSS tended to overclassify malignancy risk, leading to an increased recommendation for FNA (37.3% with AI vs. 30.7% without AI). This suggests that transferring AI models trained in high-risk settings to lower malignancy prevalence environments may compromise their performance, leading to excessive referrals without clear clinical benefits. Therefore, it is crucial to adapt and train AI algorithms specifically for these clinical settings to optimize their utility and prevent over-referrals and associated risks. As shown in Table 3, agreement analysis demonstrated low concordance in all ACR-TIRADS and ATA characteristics between the GE and AI-DSS. However, when comparing TNC subspecialist evaluations with AI-DSS, moderate-to-high agreement was observed for most ultrasound features. This level of agreement is comparable to that observed between GE and TNC subspecialist and is consistent with previous studies [6, 7, 17]. From our perspective, the increased agreement when analyzing nodules of intermediate-to-high suspicion that were considered FNA candidates by GE supports the notion of an AI-DSS adaptability issue in low malignancy prevalence settings.

Finally, the use of AI-DSSs is inherently influenced by the risk stratification scale applied. In our study, 31.9% of thyroid nodules were reclassified depending on whether the ATA 2015 or ACR TI-RADS system was used, as shown in Fig. 1. These discrepancies between classification systems are well-documented in the literature [18], and they can significantly affect both the diagnostic performance and clinical recommendations—even when such recommendations are generated by the AI itself.

This study represents the first analysis of AI-DSS use in a real-world clinical setting characterized by low malignancy prevalence. Despite the limited sample size, the statistical power calculation ensured statistical significance and the robustness of the findings, accurately reflecting thyroid nodule evaluation in general practice. The random selection of thyroid nodules and participation of non-thyroid subspecialist endocrinologists helped minimize bias and enhance the study’s external validity. Furthermore, our findings align with published literature, reinforcing their reliability. Finally, the absence of malignant thyroid pathology in the analyzed sample may be considered a limitation, as it does not allow for the evaluation of the diagnostic performance of the AI-DSS. However, this reflects the real-world scenario of thyroid nodule assessment in low-malignancy settings, where the prevalence of thyroid nodule is high and ultrasound is used exclusively as a screening tool.

In conclusion, non-adjunct AI-DSS use did not significantly improve risk stratification or reduce hypothetical referrals for additional studies to subspecialized units in a low-complexity thyroid nodule setting. The system tended to overestimate risk, potentially leading to unnecessary procedures. Additionally, this study found low agreement between AI-DSS and general endocrinologists in various ultrasound descriptors, highlighting the need for further optimization of AI tools in low-prevalence environments.

## Data Availability

No datasets were generated or analysed during the current study.
